# Supplementary material for: Active machine learning model for the dynamic simulation and growth mechanisms of carbon on metal surface
Source: Nat Commun. 2024 Jan 6;15:344. doi: 10.1038/s41467-023-44525-z (PMC10771457; doi:10.1038/s41467-023-44525-z)
Supplement: Supplementary file 1 — Supplementary Information [file 41467_2023_44525_MOESM1_ESM.pdf]

Supplementary Information for "Active Machine Learning-Driven Model for the Dynamic Simulation and Growth Mechanisms of Carbon on Metal Surface"

Di Zhang[1,3]*, Peiyun Yi[1,2], Xinmin Lai[1,2], Linfa Peng[1,2*], Hao Li[3*]

1 State Key Laboratory of Mechanical System and Vibration, Shanghai Jiao Tong University, Shanghai 200240, P.R. China

2 Shanghai Key Laboratory of Digital Manufacture for Thin-walled Structures, Shanghai Jiao Tong University, Shanghai 200240, P.R. China

3 Advanced Institute for Materials Research (WPI-AIMR), Tohoku University, Sendai 980-8577, Japan

*Corresponding author Email:

zhangdi2015@sjtu.edu.cn (D.Z.)

penglinfa@sjtu.edu.cn (L.F.P.)

li.hao.b8@tohoku.ac.jp (H.L.).

Supplementary Method 1. Hybrid Molecular-dynamics and time-stamped force-biased Monte Carlo methods

MD simulations and tfMC simulations were alternately conducted to simulate carbon atoms' deposition and relaxation processes. Originally known as the uniform-acceptance force-bias Monte Carlo (UFMC) method,[1] tfMC differs from molecular dynamics (MD) algorithms by employing a force-bias probabilistic description of atomic motion. This approach has demonstrated success in replicating long-term events that occur during phase transitions, surface diffusion, and growth.[1, 2, 3, 4]. In 2012, Mees *et al.*[4] introduced a statistically relevant time step per UFMC iteration, resulting in a significant speed-up compared to MD simulations. As a result, the UFMC method became known as the time-stamped force-bias Monte Carlo (tfMC).

During tfMC simulations, each step, all atoms in the selected group are displaced using the stochastic tfMC algorithm[1], which is designed to sample the canonical (NVT) ensemble at the temperature. Although tfMC is a Monte Carlo algorithm and thus strictly speaking does not perform time integration, it is similar in the sense that it uses the forces on all atoms in order to update their positions. In a single tfMC iteration step, each atom undergoes stochastic displacements in the three Cartesian directions (δ_{v}, $v=x, y, z$), with a user-selectable parameter Δ/2 defining the range. The displacements (δ_v) are normalized by Δ/2 to ensure they fall within the range of -1 to 1, as shown in the equation (1):

$$\xi_v = \frac{\delta_v}{\Delta / 2} \tag{1}$$

To generate a value for ζ_v, a uniform random number η_v is used, which is sampled from the interval [0, 1], as shown in the equation (2):

$$\xi_v = \frac{2k_B T}{F_v \Delta / 2} \ln[\eta_v (e^{\left|\frac{F_v \Delta/2}{2k_B T}\right|} - e^{-\left|\frac{F_v \Delta/2}{2k_B T}\right|}) + e^{-\left|\frac{F_v \Delta/2}{2k_B T}\right|}] \tag{2}$$

where F_v is the v component of the force **F** acting on the atom under consideration, k_B the Boltzmann constant, and T the temperature chosen for the tfMC simulation. The tfMC algorithm described above has been successfully implemented in the LAMMPS program[5].

In the tfMC method, two critical parameters, temperature (T) and maximum allowed displacement (Δ), need to be carefully chosen. For T, it is important to select a realistic and physically meaningful value. The parameter Δ is determined through a series of simulation tests at different deposition temperatures, following the criterion proposed by Timonova *et al.*[1]. This criterion ensures that a perfect crystal remains perfect after tfMC simulation and a short MD equilibration. The parameter values Δ = 0.18 Å and T = 573 K were carefully determined and utilized in our previous work[2] and were also employed in this study.

The main purpose of the energy/velocity rescaling walls is to prevent the generation of artificial simulation results caused by the repetitive entry of impinging atoms' excessive kinetic energy through periodic boundary conditions[6]. In reality, the surface of a material is considered infinite at the microscopic level. However, in simulations, we use periodic boundary conditions to mimic an infinitely large surface on a finite-sized box. In real deposition processes, the kinetic energy from impinging atoms dissipates into the surrounding environment. To emulate this energy dissipation, we implemented an energy/velocity rescaling wall, as depicted in Supplementary Figure 1a and Supplementary Figure 1b (representing the side and top view, respectively). The axis of symmetry of this wall is perpendicular to the deposition surface. This wall is designed to modulate the kinetic energy's dissipation, thereby ensuring simulations that are more physically representative and meaningful.

Supplementary Figure 1 clearly illustrates the temperature profile during the MD/tfMC deposition simulation. As observed, the velocity/energy walls in the deposition system play a

crucial role in maintaining stability. Without them, the excessive energy from impinging atoms would repeatedly enter the system, leading to instability and unrealistic high thermal spike.

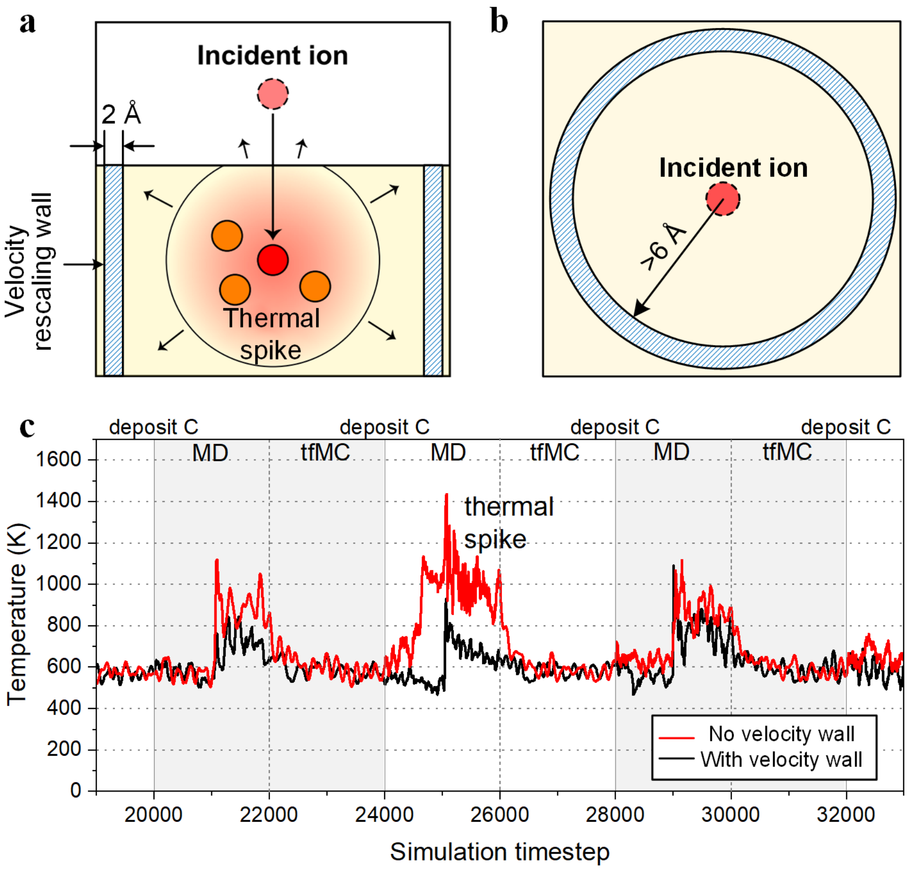

Supplementary Figure 1. The impact of the energy/velocity wall. (a) Side view, (b) Top view, and (c) A comparative analysis of thermal spikes in deposition simulations, highlighting differences with and without the energy/velocity rescaling walls. Source data are provided as a Source Data file.

Supplementary Method 2. DFT calculations and Climbing-image NEB calculations

All the energies and forces of the structures in the training database are calculated by the QUICKSTEP scheme in CP2K[7, 8]. Electronic wave functions were described at the Gamma point using a mixed-basis scheme with Goedecker-Teter-Hutter (GTH) pseudopotentials[9] and a cutoff and the relative cutoff energy of 300 Ry and 60 Ry. Shorter range Double-ζ quality basis sets optimized for GTH Perdew-Burke-Ernzerhof (PBE)[10] pseudopotential with 4 and 11 valence electrons were used for the carbon and copper atoms. Dispersion corrections are included by the well-established Grimme's D3 method[11].

The CI-NEB calculations were performed using the following specifications: We used a total of 4 or 8 replica geometries along the path with a spring constant of 0.001 to restrain the replicas. Convergence criteria were set as follows: the maximum displacement (MAX_DR) was limited to 0.02 Å, the maximum force (MAX_FORCE) to 0.05 eV $Å^{-1}$, the root mean square displacement (RMS_DR) to 0.01 Å, and the root mean square force (RMS_FORCE) to 0.001 eV $Å^{-1}$. The optimization algorithm employed was Direct Inversion in the Iterative Subspace (DIIS), with a maximum of 1000 optimization steps and 3 DIIS vectors. The BAND_TYPE used for the calculations was Climbing-image NEB.

Supplementary Method 3. Validation of the hyper-parameters and descriptors for the CGM-MLP

In Supplementary Figure 2, we present the force Mean Absolute Error (MAE) as a function of hyper-parameters (sparse points and SOAP cutoff) and descriptor parameters (n_{max} and l_{max}) specifically for the Cu-containing system. We generated training sets consisting of Cu(111) slabs deposited with varying numbers of carbon atoms (a total of 1085 structures) using the CGM-MLP training framework. Additionally, we selected testing sets from the structures held-out during the training process (a total of 500 structures). The results demonstrate that the hyper-parameters and descriptor parameters we employed, namely Sparse Points=9000, SOAP cutoff=3.7 Å, l_{max}=4, and n_{max}=12, are capable of achieving the same level of predictive force accuracy as observed with the GAP-20 potential.

The force errors are defined as the MAE of the force components along three directions (x, y, and z) for all structures in the training set, as shown in the following equation (3):

$$F_{MAE} = \sum_{j=1}^{S} \frac{\sum_{i=1}^{N_j} \left|F_{x,i}^{DFT} - F_{x,i}^{MLP}\right| + \left|F_{y,i}^{DFT} - F_{y,i}^{MLP}\right| + \left|F_{z,i}^{DFT} - F_{z,i}^{MLP}\right|}{3N_j} \Bigg/ S \tag{3}$$

where $F_{x,i}$, $F_{y,i}$, and $F_{z,i}$ represent the x, y, and z components of the force. These components are obtained from both DFT calculations and other force fields, which encompass the MLP and classical empirical potentials. N represents the total number of atoms within a given structure, while S denotes the total number of structures present in the training set.

The energy errors are quantified as the MAE between the energies derived from both DFT calculations and the MLP model, as shown in the equation (4).

$$E_{MAE} = \sum_{i=1}^{S} \frac{\left|E_i^{DFT} - E_i^{MLP}\right|}{S} \tag{4}$$

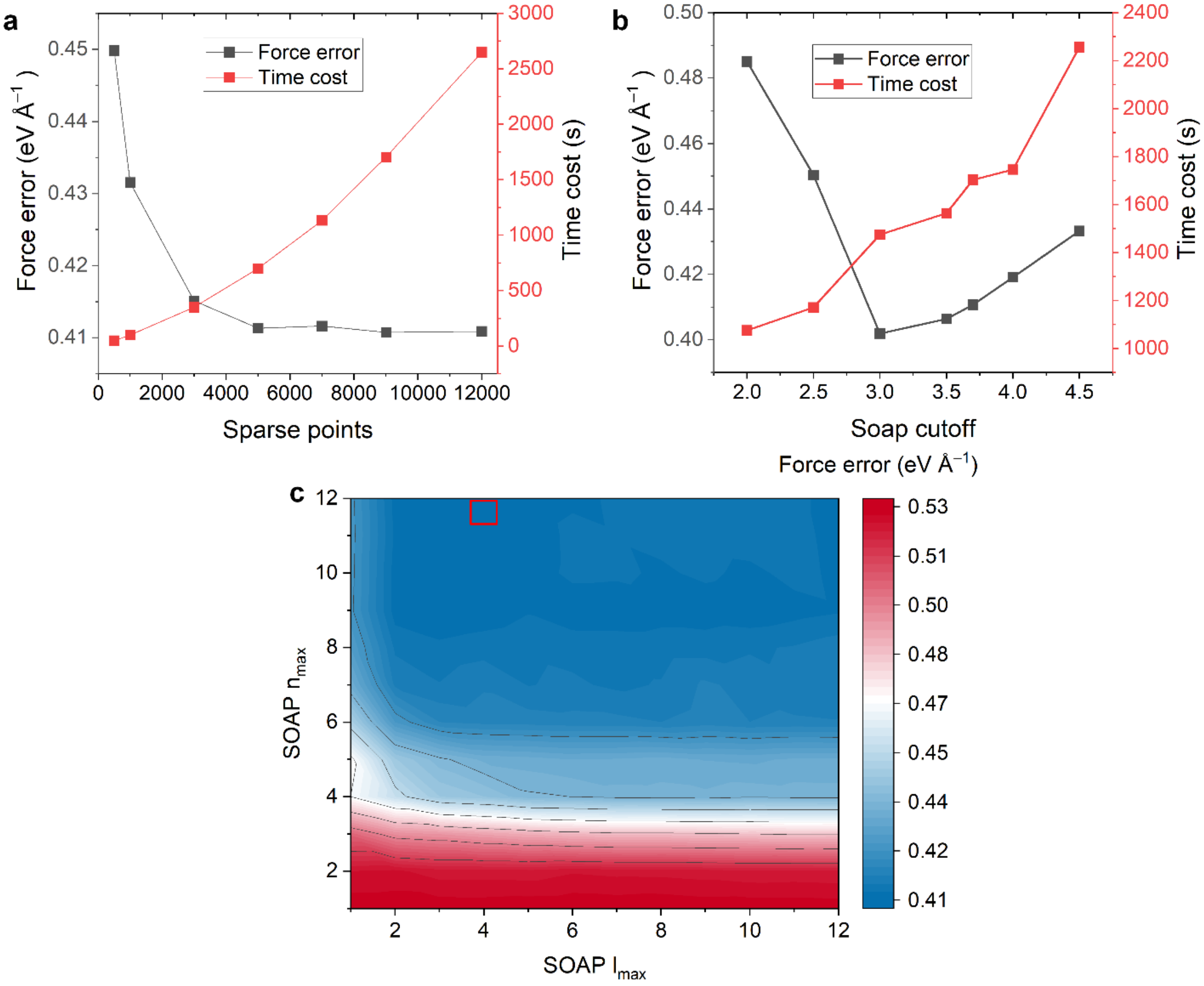

Supplementary Figure 2. Hyper-parameter and descriptor tests of the Deposited-C@Cu(111) set. (a) Sparse point tests. (b) smooth overlap of atomic positions (SOAP) cutoff test. (c) n_{max} and l_{max} test. The hyper-parameters and descriptor parameters we employed, namely Sparse Points=9000, SOAP cutoff=3.7 Å, l_{max}=4, and n_{max}=12, are capable of achieving the same level of predictive force accuracy as observed with the Gaussian Approximation Potential (GAP)-20 potential. Source data are provided as a Source Data file.

Supplementary Method 4. Testing the Screening Parameters (*S^{max}* and *S^{ave}*) for *D^{max}* and *D^{ave}*

The environmental similarity of two atoms is evaluated using the Euler distance between their respective SOAP vectors. During the evaluation of a candidate structure, each atom is assigned the closest distance to the existing training dataset. Atoms in the candidate dataset with larger closest distances are given higher priority for inclusion in the training set. The D^{ave} value represents the average closest distance among all atoms in the candidate structure, while the D^{max} value represents the maximum closest distance. For enhanced clarity, we have provided Python code for calculating D^{ave} and D^{max} on the website: https://github.com/sjtudizhang/CGM-MLP/Calculate_similarity.py[12]. if a new structure's D^{ave} or D^{max} exceeds the specified screening parameter (S^{max} or S^{ave}), the new structure will be included in the training set.

To evaluate the effectiveness of the screening parameters, we selected a subset of structures from the MD/tfMC simulations using different values of N_f, S^{max}, and S^{ave}. Our approach here is based on an understanding: Structures chosen during the on-the-fly training process by more lenient screen parameters (larger S^{max} and S^{ave}) will undoubtedly be selected by stricter ones (smaller S^{max} and S^{ave}). For instance, in Supplementary Table 1, the "Deposited-Carbon@Cu" group was chosen using the strictest screen parameters (i.e., S^{max}, S^{ave}=0.06). Based on this set of structures, we employed more lenient screen parameters (e.g., S^{max}=0.08~0.40, S^{ave}=0.08) to derive different training sets. These sets, in turn, were utilized to train various MLPs and the results of energy MAE are shown in Supplementary Figure 3 and Supplementary Figure 4. A significant benefit of this method is the elimination of potential influence due to the randomness of MD/tfMC simulations. Meanwhile, 468 structures were randomly selected from the held-out structures to serve as the testing sets. By increasing N_f and lowering S^{max}, as shown in Supplementary Figure 3a and b, the CGM-MLP exhibits improved energy/force accuracies and a significant improvement

compared to classical empirical potentials, such as COMB3[13] and ReaxFF[14]. Specifically, the energy and force MAE of the CGM-MLP trained with $N_f = 20$ and S^{ave}, $S^{max} = 0.08$ converge to approximately 0.013 eV and 0.43 eV Å^{-1}, respectively (Supplementary Figure 3c).

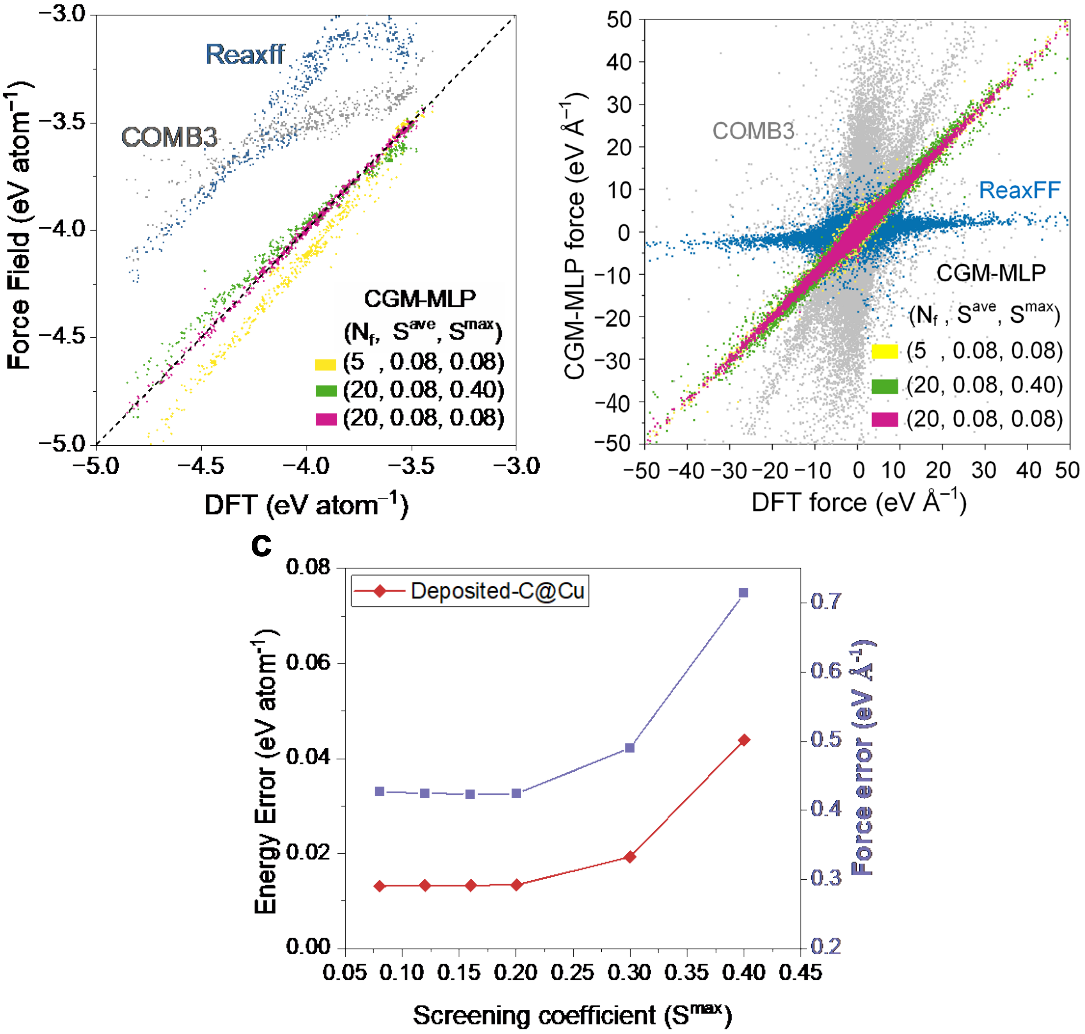

Supplementary Figure 3. Energy/force correlation plots in production runs by using different quality control parameters, namely N_f, $S^{max,}$ and S^{ave}. N_f is the number of structures sampled for each deposited carbon atom. S^{max} and S^{ave} are the thresholds for the maximum and average SOAP distances, D^{ave} and D^{max}. (a) Energy and (b) force correlation plots. (c) Production runs by using different S^{max}, with S^{ave} fixed at a low value of 0.08. Source data are provided as a Source Data file.

To test the application of S^{max} and S^{ave} in different types of structures, we have generated a series of training and testing datasets for various structure types, such as graphite crystals, amorphous carbon, and deposited carbon on the Cu(111) surface. Detailed information on dataset selection can be found in Supplementary Table 1. Additionally, we have also made the entire dataset available for evaluating the screening parameter *S* on the GitHub repository: https://github.com/sjtudizhang/CGM-MLP/testing_screening_paramters[12].

Supplementary Table 1. The testing and training sets used for testing the screen parameters

Structure Group	Training Set	Testing set	File Name
Graphite	40 graphite structures with different lattice constants ranging from 2.0 to 3.2 Å, with a 0.03 Å increment	20 randomly generated graphite crystal structures with lattice constants ranging from 2.0 to 3.2 Å	Graphite_Training_Set.xyz<br>Graphite_Testing_Set.xyz
Amorphous Carbon	2558 structures selected from the GAP-20 database[15]	493 structures available from the GAP-20 database[15], excluding any structures present in the training set.	Amorphous_Carbon_Training_Set.xyz<br>Amorphous_Carbon_Testing_Set.xyz
Carbon-Cluster@Cu	588 structures selected from the AIMD simulation of the Cu(111) slab, including both the C1-C18 clusters on the Cu(111) slab	192 structures were uniformly selected from the AIMD simulation, excluding any structures that are part of the training set.	Carbon_Cluster_Training_Set.xyz<br>Carbon_Cluster_Testing_Set.xyz
Deposited-Carbon@Cu	1090 structures uniformly selected from the MD/tfMC simulation during the training process of CGM-MLPs	468 structures uniformly selected from the MD/tfMC simulation, excluding any structures that are part of the training set.	Deposited_Carbon_Training_Set.xyz<br>Deposited_Carbon_Testing_Set.xyz

Following the creation of the training and test sets, we proceeded to screen the training sets using various screen parameters (S^{max}) ranging from 0.06 to 0.40, while the S^{ave} was fixed at 0.08. The screened training sets were then employed to train MLPs. Subsequently, these trained MLPs were used to calculate the energy MAE for the corresponding test sets. The results of these calculations are presented in Supplementary Figure 4. It is worth noting that the similarity measure can imply different errors for different training sets. Generally, the more drastic the variations in the potential energy surface, while achieving the same level of predictive accuracy, the smaller the value of S^{max} is required. In different system applications, it is necessary to test various systems based on the precision requirements and ultimately select the appropriate value of S^{max} and S^{ave}. In this study, it was found that when the value of S^{max} is below 0.1, all predictive errors remain below 0.05 eV atom^{-1}. Therefore, we selected S^{max}, S^{ave} = 0.08 as the parameters to train the CGM-MLP in this work.

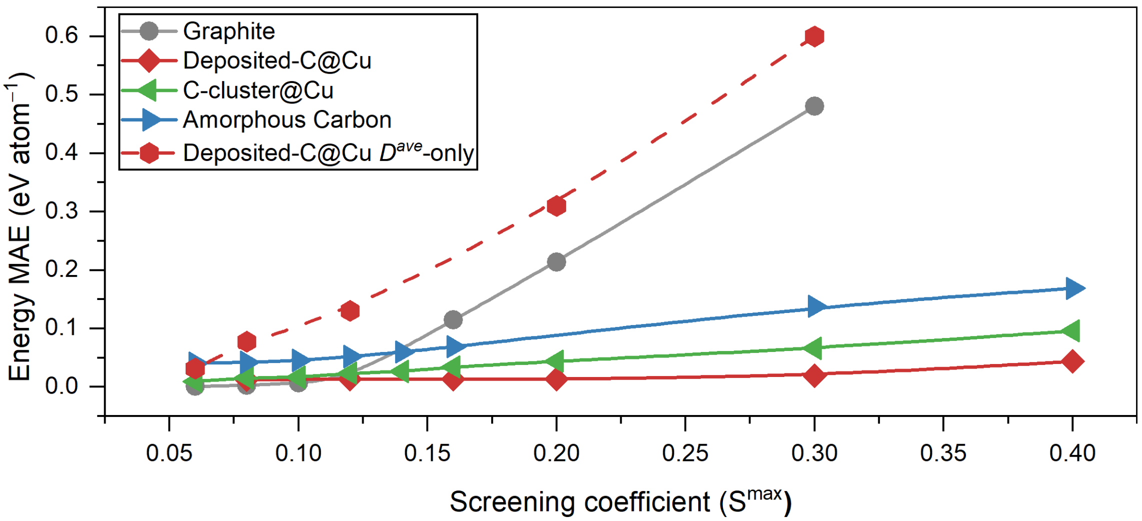

Supplementary Figure 4. The screen parameter S^{max} and corresponding predictive accuracies for different structures, with a fixed low S^{ave} value of 0.08. Source data are provided as a Source Data file. S^{max} is the threshold of maximum SOAP distances for controlling whether a new structure will be added into training sets.

In Supplementary Figure 4, we also conducted tests to examine the relationship between S^{ave} and predictive accuracy when using only D^{ave} to filter the candidate structure dataset. As mentioned before, relying only on the configuration-averaged metric for selecting new structures could omit structures that exhibit significant variations only in the local areas surrounding the deposition atom. To address this limitation, we propose not only using the configuration-averaged similarity metric (D^{ave}) but also including the structure with a single atom displaying the most distinct environment (D^{max}). In Supplementary Figure 4, by simultaneously using both D^{ave} and D^{max} as filtering criteria, as compared to solely utilizing D^{ave} (the red dashed line), we achieve the same accuracy level with a more relaxed screen parameter.

Supplementary Discussion 1. Test of Long-range Interactions for Metal-Carbon Systems

To elucidate the influence of long-range interactions the carbon structures formed on metallic surfaces in our simulations, we constructed seven distinct data sets, details of which are presented in Supplementary Figure 5. The first set (Set#1) comprises 2000 pure-carbon structures, which were randomly sampled from GAP20. For the data derived from active learning and the final simulation structures, we categorized them based on the number of carbon atoms into two groups: those associated with a Cu slab containing fewer than 10 carbon atoms and those with more than 10 carbon atoms (Supplementary Figure 5a). To evaluate the impact of long-range interactions across various metal-carbon configurations, we also created two groups: Ti and Cr slabs, each with fewer than 10 carbon atoms, both of which were derived from actively learned data.

Supplementary Figure 5b displays force correlations for three groups: GAP20 structures, $C_{1\sim10}$ on Cu, and $C_{10\sim100}$ on Cu. GAP20 structures exhibit the strongest long-range interactions. Initially, carbon monomers primarily interact with the Cu(111) surface, reducing both long-range interactions and the force mean absolute error (MAE). As deposition progresses, carbon atoms accumulate on the Cu surface and form clusters. This resurgence in long-range interactions can influence the force accuracy of machine learning potentials (MLPs). This trend persists in the test of final Cu-C simulation structures. In contrast, the Cr-C and Ti-C systems, with fewer carbon atoms, achieve a force accuracy of 0.08 eV $Å^{-1}$, indicating minimized long-range interaction effects (Supplementary Figure 5c).

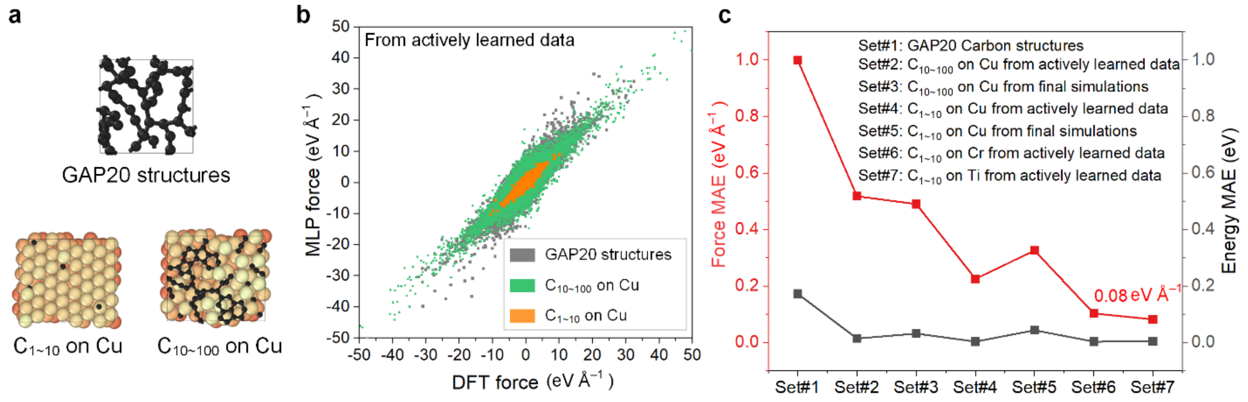

Supplementary Figure 5. Test of long-range interactions for metal-carbon systems. (**a**) Three examples of the testing structural groups. (**b**) Force correlations of Gaussian Approximation Potential (GAP)-20 structures, $C_{1\sim10}$ on Cu, and $C_{10\sim100}$ on Cu. The subscripts in $C_{1\sim10}$ and $C_{10\sim100}$ indicate the range of the number of carbon atoms on the Cu surface. (**c**) Force and energy mean absolute error (MAE) of seven different data sets, primarily encompassing varying numbers of carbon atoms and different metal elements. Specific labels can be found in the inset provided. Source data are provided as a Source Data file.

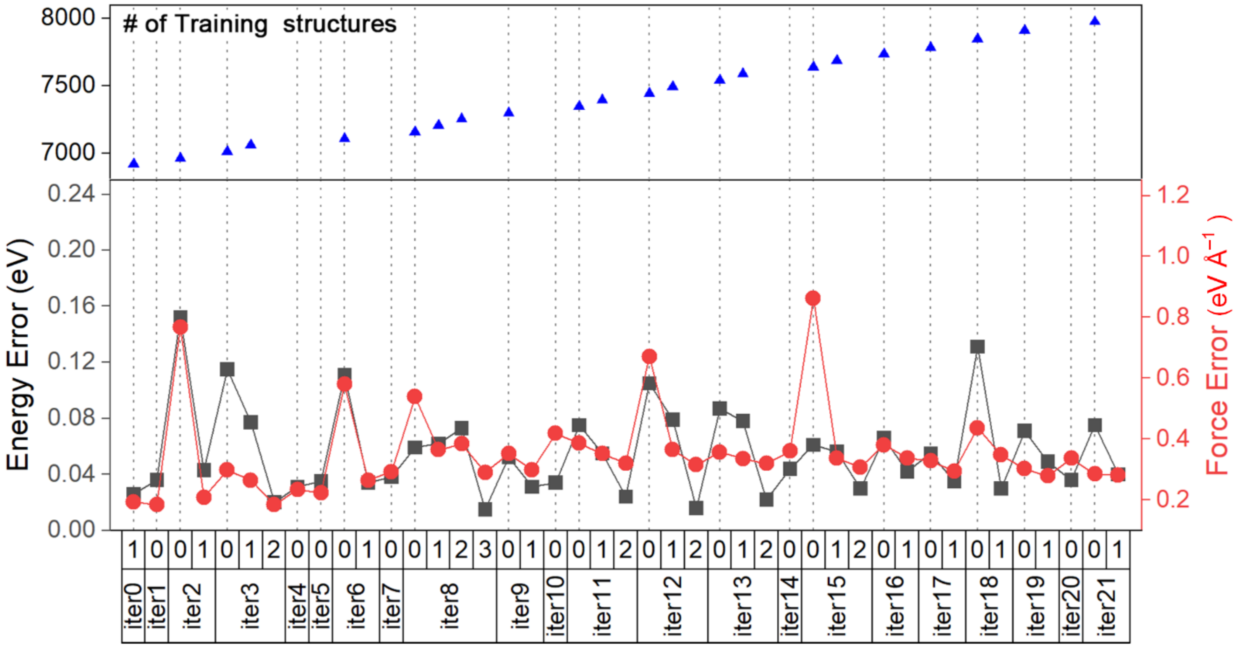

Supplementary Figure 6. Energy and force errors over 20 iterations during the training process. A criterion of less than 0.05 eV atom^{-1} for energy and less than 0.5 eV Å^{-1} for force (highlighted by the horizontal dashed line) determines whether newly generated structures are integrated into the training set or if the deposition of carbon atoms can continue without additional training. With N_f = 20, S^{max}, S^{ave} = 0.08, approximately 40~60 structures will be added to the training set. N_f, S^{max} and S^{ave} retain the same meanings as defined in Supplementary Figure 3. Source data are provided as a Source Data file.

Supplementary Discussion 2. The Effect of Adsorbed Cu Atoms on the Nucleation Stability

To calculate the minimum activation energy required by moving a surface copper atom to a hollow adsorbed site, we employed the CI-NEB method combined with DFT and the CGM-MLP to calculate the potential energy as a function of the reaction coordinate. Supplementary Figure 7a presents that the activation energy is about 2.0 eV, and this energy from the CGM-MPL is about 0.5 eV higher than that from DFT. This surmountable activation energy offers opportunities to transition from surface Cu atoms to adatom Cu atoms. In addition, Fig.3c shows the binding energy of a ring-like C8 cluster on the Cu(111) surface as a function of the number of passivated Cu atoms. The binding energies were calculated by the equation,

$$E_{8C*} = E_{8C}^{\text{tot}} - E_{8C} - E_{*} \tag{5}$$

where E_{8c}^{tot} is the total energy of the C8 cluster and substrate, and E_{8C} is the energy of the C8 cluster in a vacuum. E_{*} is the energy of the substrate with a different number of Cu adatoms. It can be seen that the greater number of Cu adatoms, the more stable the 8C carbon rings are.

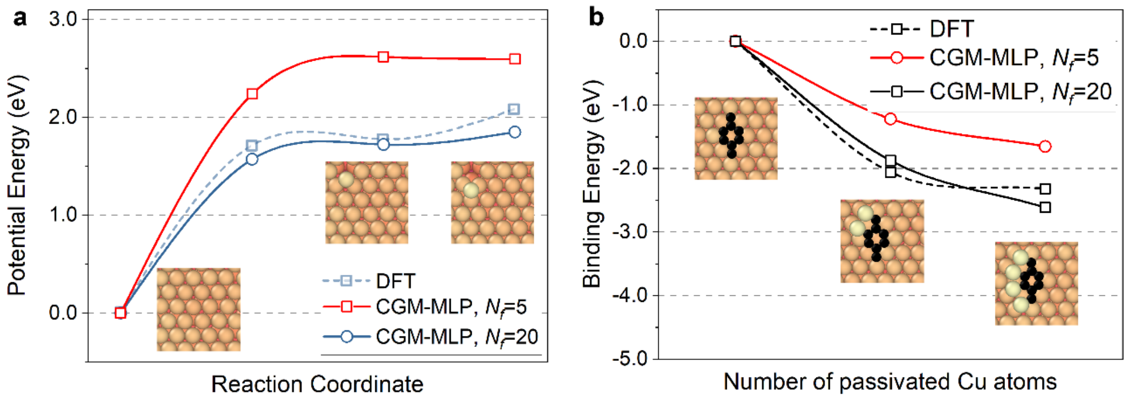

Supplementary Figure 7. The role of Cu adatoms in stabilizing the early stages of graphene nucleation. (a) Potential energies tracked during the off-surface movement of a singular Cu atom. (b) The binding energy of the C_8 ring on the Cu(111) surface in the presence of varying Cu adatoms. Source data are provided as a Source Data file.

To ensure a transparent and comprehensive error assessment, we've presented the total reaction barriers in Supplementary Figure 8, which corresponds to Fig. 4 in the main manuscript. The absolute error for the reactions in Supplementary Figure 8 ranges from 0.1 eV to 0.49 eV. These deviations in total energy may arise from three primary factors: the precision of CGM-MLP, long-range interactions, and the number of atoms involved in the reactions. While total energy barriers can indicate the overall predictive accuracy for a reaction, it's worth considering that reactions involving more atoms could potentially exhibit higher total energy errors. Nonetheless, when viewed on a per-atom basis, these errors may be relatively small, thereby ensuring that the predicted reaction pathway remains highly reliable in practice. Therefore, we used the per-atom energy barriers in the main text.

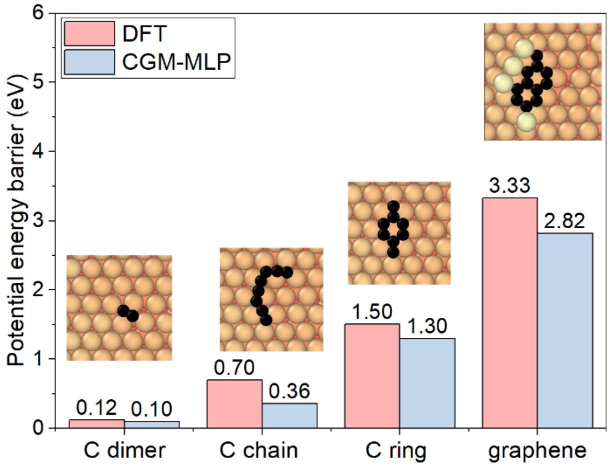

Supplementary Figure 8. Comparison of total energy barriers as calculated by DFT and carbon-growth-on-metal machine-learning potential (CGM-MLP).

Supplementary Discussion 3. Empirical Cu-C potentials for the Deposition Simulations

In Fig.1c of the main text in this work, we have compared the energy and force errors calculated from a ReaxFF[14], a charge-optimized many-body potential (i.e., COMB3)[13], and CGM-MLP. For comparisons, we also used these two empirical Cu-C potentials to simulate the carbon growth on Cu(111) surface by employing the hybrid MD/tfMC method. Supplementary Figure 9 illustrates the results of the COMB3-based simulations, revealing that during the initial stages, the deposited carbon atoms are capable of diffusing into the sublayer of the Cu(111) surface, as observed in Fig. 3 of the main manuscript. However, it is notable that the carbon atoms tend to form metal carbide rather than graphitic structures on the Cu(111) surface, and even at the end of the simulations, no graphitic structures are observed. This outcome contradicts both experimental observations and DFT calculations. Consequently, it suggests that the COMB3 potential may not be suitable for accurately simulating the growth of graphene on metal surfaces.

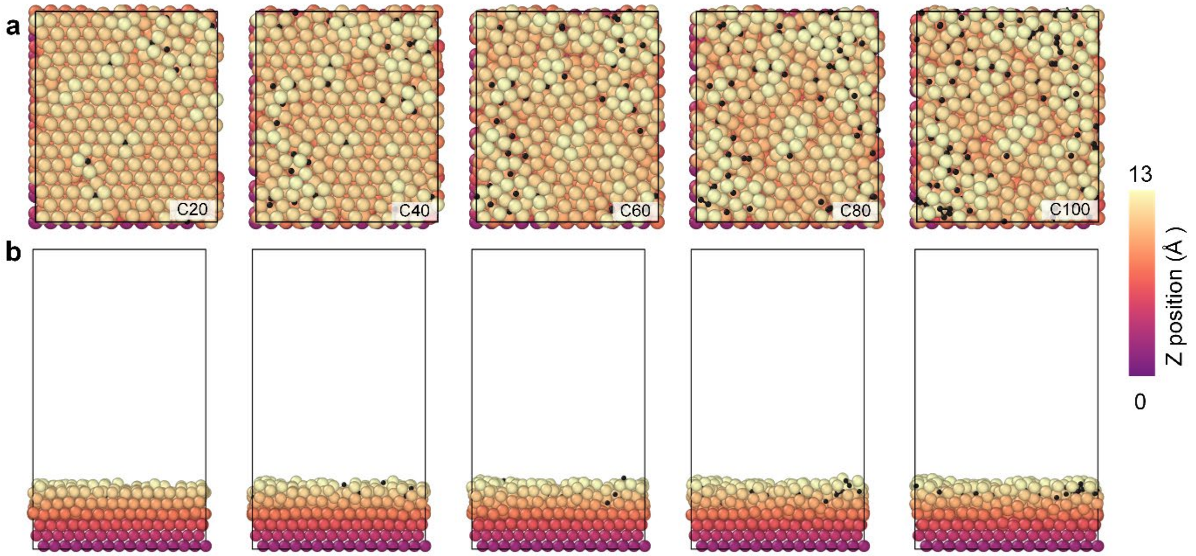

Supplementary Figure 9. Charge-optimized many body (COMB3)-based[13] hybrid molecular dynamics and time-stamped force-biased Monte Carlo (MD/tfMC) simulations of carbon growth on Cu(111) surface. (a) Top view (b) Left view.

Supplementary Figure 10 illustrates the results of a ReaxFF-based[14] deposition simulation of carbon atoms on the Cu(111) surface, where the incident energy is set at 5.0 eV. During the deposition process, it is observed that both carbon and a small portion of copper atoms diffuse toward the vacuum region. The vacuum region exhibits clusters of carbon and copper, but no graphene nucleation on Cu(111) surface is observed. As depicted in Fig. 1c in the main text, the predicted forces by the ReaxFF are only accurate within limited phase space. In the case of deposition simulations, where many states deviate significantly from equilibrium, ReaxFF fails to provide satisfactory results when applied to such simulations.

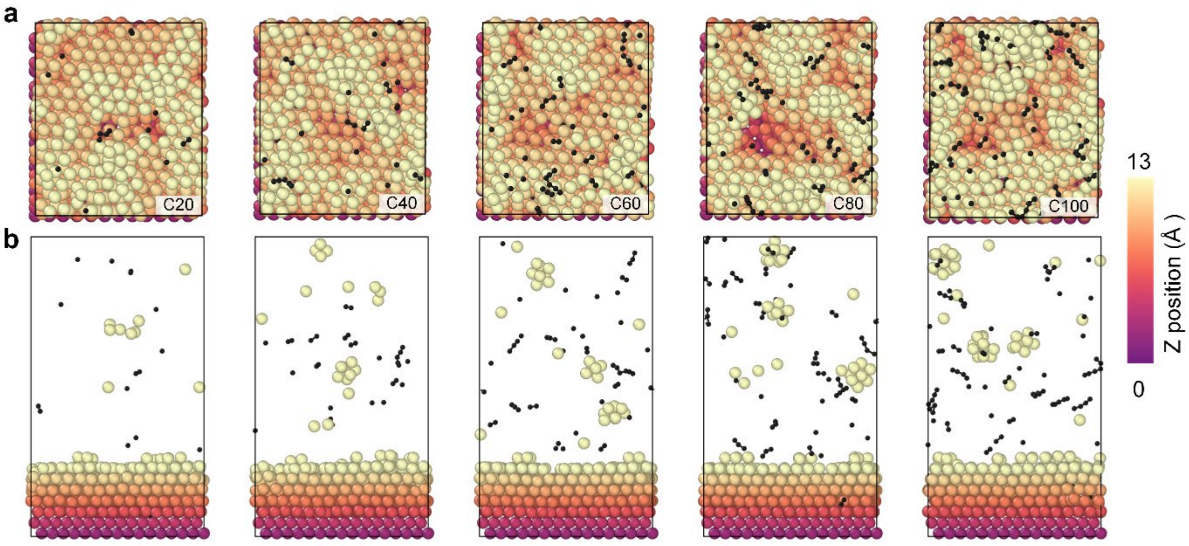

Supplementary Figure 10. Reactive force field (ReaxFF)-based[16] hybrid molecular dynamics and time-stamped force-biased Monte Carlo (MD/tfMC) simulations of carbon growth on Cu(111) surface. (a) Top view (b) Left view.

Supplementary Discussion 4. Effects of surface oxygen contamination and detailed simulation processes

The presence of oxygen or hydrogen contamination can significantly influence the nucleation and epitaxial growth of graphene on Cu(111). To demonstrate the transferability of our CGM-MLP training framework, we have extended our method to construct a ternary Cu-C-O MLP. Building upon the Cu-C MLP, we initiated the training process using an O-contaminated Cu(111) surface (Supplementary Figure 11a) as the starting point. The quality control parameters for the training process were consistent with those used for training the Cu-C MLPs. After 25 iterations of training, additional oxygen-containing 1723 structures were added to the training set, which was then employed to fit the Cu-C-O MLP.

Using the Cu-C-O MLP-based MD/tfMC method, we performed deposition simulations of carbon on O-contaminated Cu(111). At the initial stage (Supplementary Figure 11b), the deposited carbon atoms exhibited a preference for binding with surface oxygen, forming CO molecules. These CO molecules were observed to diffuse rapidly on the Cu(111) surface or escape into the vacuum region. Due to the binding with oxygen, fewer carbon atoms penetrated the sublayer of Cu(111). As the deposition process continued, short carbon chains were observed on the Cu(111) surface, but with the presence of oxygen contamination, the formation of C-Cu-O bridges was also generally evident (Supplementary Figure 11c). As discussed in the main text, the presence of C-Cu bridges is crucial for the formation of initial carbon rings, However, the C-Cu-O bridges may hinder the combination of carbon chains to form potential carbon rings. Additionally, oxygen atoms were observed to bind with the short carbon chains, acting as terminal groups and further inhibiting the formation of small carbon rings. After depositing 100 carbon atoms, the absence of oxygen contamination led to the formation of small graphene islands on Cu(111) (Fig. 5a).

However, in the presence of oxygen, the final structure consisted of cross-linked carbon chains (Supplementary Figure 11e). Therefore, the presence of C-Cu-O bridges and terminated oxygen atoms significantly reduced the initial nucleation rate of graphene on Cu(111), which is in agreement with previous experimental observations[17].

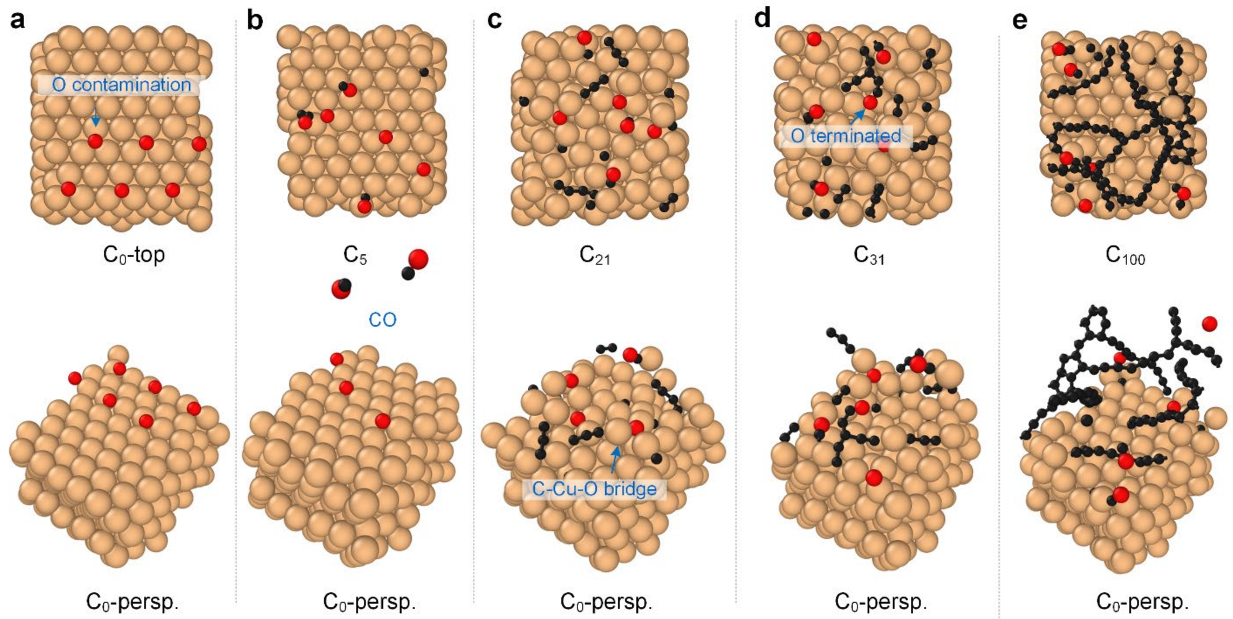

Supplementary Figure 11. The significant effect of oxygen surface contamination on the nucleation process. The presence of C-Cu-O bridges and oxygen terminal groups on the Cu(111) surface significantly reduces the nucleation rate of carbon, resulting in the formation of cross-linked carbon chains instead of small graphene islands.

To ensure the reproducibility of MLPs in practical applications, we repeatedly conducted MD/tfMC simulations under various carbon atom incident energies by using different random seeds (Supplementary Figure 12). This allowed us to replicate and validate the performance of MLPs.

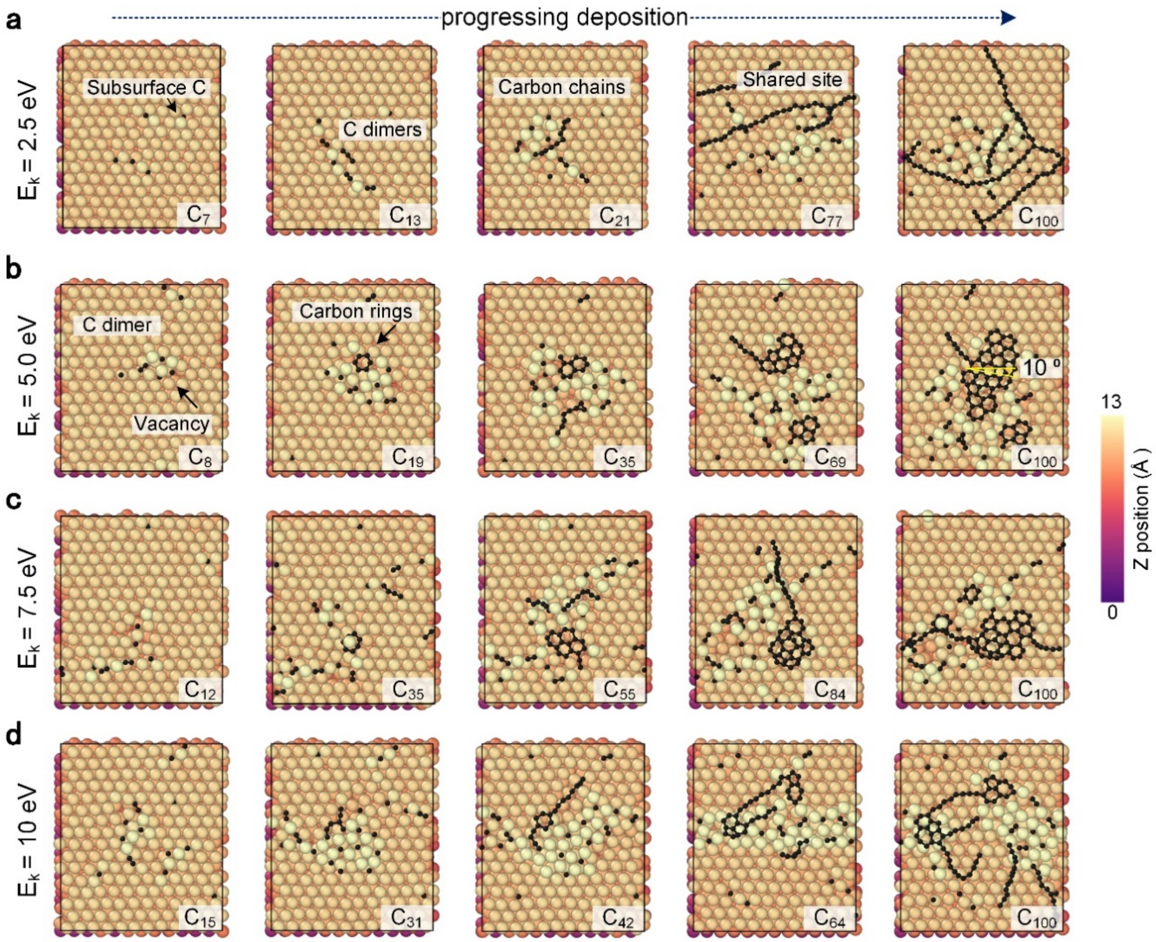

Supplementary Figure 12. Repeated CGM-MLP driven simulations of graphene growth on Cu(111) with different incident kinetic energies (E_k), *i.e.*, (a) 2.5 eV, (b) 5.0 eV, (c) 7.5 eV, and (d) 10 eV. All simulated results in this work were visualized with the help of OVITO[18]. Carbon atoms are colored black, and copper atoms are color-coded according to their height coordination. For the labels "C_X", "X" denotes the number of deposited carbon atoms.

Supplementary References

1. Timonova M, Groenewegen J, Thijsse BJ. Modeling diffusion and phase transitions by a uniform-acceptance force-bias Monte Carlo method. *Physical Review B* **81**, 144107 (2010).

2. Zhang D, Peng L, Li X, Yi P, Lai X. Controlling the Nucleation and Growth Orientation of Nanocrystalline Carbon Films during Plasma-Assisted Deposition: A Reactive Molecular Dynamics/Monte Carlo Study. *Journal of the American Chemical Society* **142**, 2617-2627 (2020).

3. Neyts EC, van Duin ACT, Bogaerts A. Formation of single layer graphene on nickel under far-from-equilibrium high flux conditions. *Nanoscale* **5**, 7250-7255 (2013).

4. Mees MJ, Pourtois G, Neyts EC, Thijsse BJ, Stesmans A. Uniform-acceptance force-bias Monte Carlo method with time scale to study solid-state diffusion. *Physical Review B* **85**, 134301 (2012).

5. Thompson AP*, et al.* LAMMPS - a flexible simulation tool for particle-based materials modeling at the atomic, meso, and continuum scales. *Computer Physics Communications* **271**, 108171 (2022).

6. Marks NA, Cover MF, Kocer C. Simulating temperature effects in the growth of tetrahedral amorphous carbon: The importance of infrequent events. *Applied Physics Letters* **89**, 131924 (2006).

7. Kühne TD, Krack M, Mohamed FR, Parrinello M. Efficient and Accurate Car-Parrinello-like Approach to Born-Oppenheimer Molecular Dynamics. *Physical Review Letters* **98**, 066401 (2007).

8. VandeVondele J, Krack M, Mohamed F, Parrinello M, Chassaing T, Hutter J. Quickstep: Fast and accurate density functional calculations using a mixed Gaussian and plane waves approach. *Computer Physics Communications* **167**, 103-128 (2005).

9. Goedecker S, Teter M, Hutter J. Separable dual-space Gaussian pseudopotentials. *Physical Review B* **54**, 1703-1710 (1996).

10. Perdew JP, Burke K, Ernzerhof M. Generalized Gradient Approximation Made Simple. *Physical Review Letters* **77**, 3865-3868 (1996).

11. Grimme S, Antony J, Ehrlich S, Krieg H. A consistent and accurate ab initio parametrization of density functional dispersion correction (DFT-D) for the 94 elements H-Pu. *The Journal of Chemical Physics* **132**, 154104 (2010).

12. Zhang D, Yi P, Lai X, Peng L, Li H. Active Machine Learning-Driven Model for the Dynamic Simulation and Growth Mechanisms of Carbon on Metal Surface. *Zenodo, DOI: 105281/zenodo10159885*, (2023).

13. Liang T*, et al.* Classical atomistic simulations of surfaces and heterogeneous interfaces with the charge-optimized many body (COMB) potentials. *Materials Science and Engineering: R: Reports* **74**, 255-279 (2013).

14. Monti S, Li C, Carravetta V. Reactive Dynamics Simulation of Monolayer and Multilayer Adsorption of Glycine on Cu(110). *The Journal of Physical Chemistry C* **117**, 5221-5228 (2013).

15. Rowe P, Deringer VL, Gasparotto P, Csányi G, Michaelides A. An accurate and transferable machine learning potential for carbon. *The Journal of Chemical Physics* **153**, 034702 (2020).

16. Zhou XW, Ward DK, Foster ME. An analytical bond-order potential for carbon. *Journal of Computational Chemistry* **36**, 1719-1735 (2015).

17. Hao Y*, et al.* The Role of Surface Oxygen in the Growth of Large Single-Crystal Graphene on Copper. *Science* **342**, 720-723 (2013).

18. Stukowski A. Visualization and analysis of atomistic simulation data with OVITO–the Open Visualization Tool. *Modelling and Simulation in Materials Science and Engineering* **18**, 015012 (2010).
