# Supplementary material for: Active machine learning model for the dynamic simulation and growth mechanisms of carbon on metal surface
Source: Nat Commun. 2024 Jan 6;15:344. doi: 10.1038/s41467-023-44525-z (PMC10771457; doi:10.1038/s41467-023-44525-z)
Supplement: Supplementary file 3 — Description of Additional Supplementary Files [file 41467_2023_44525_MOESM3_ESM.pdf]

Description of Additional Supplementary Files

Supplementary Movie 1:

Machine Learning Force Field Simulation of Carbon Deposition on Cu(111) Surface.

Supplementary Movie 2:

Carbon ring breakage by high-energy bombardments.
